# Comparing 17β‐estradiol and progesterone concentrations in young, physically active females: Insights from plasma versus serum analysis

**DOI:** 10.1113/EP092415

**Published:** 2025-03-12

**Authors:** Samantha N. Rowland, Mariasole Da Boit, Rachel Tan, Liam M. Heaney, Stephen J. Bailey

**Affiliations:** ^1^ Women in Sport Research and Innovation Hub, School of Sport, Exercise and Health Sciences Loughborough University Loughborough UK; ^2^ Health and Life Sciences, School of Allied Health Sciences De Montfort University Leicester UK; ^3^ Natural Science Division Pepperdine University Malibu California USA

**Keywords:** contraception, hormones, immunoassay, menstrual cycle, physiology

## Abstract

Serum measurements of 17β‐estradiol and progesterone are widely used to verify menstrual cycle status and confirm contraceptive use, often through commercially available immunoassay kits. However, no studies have investigated whether blood collection tube chemistries influence hormone concentrations in young females, despite assays permitting the use of different biofluids with similar reference ranges. In this study, venous blood was sampled from physically active females (*n* = 25) using Ethylenediaminetetraacetic acid (EDTA) and serum vacutainers, and 17β‐estradiol and progesterone concentrations were measured using competitive immunoenzymatic assays. Median plasma concentrations of 17β‐estradiol and progesterone were 44.2% (plasma 40.75 vs. serum 28.25 pg/ml) and 78.9% (plasma 1.70 vs. serum 0.95 ng/ml) higher than serum concentrations, respectively (*P *< 0.001 for both). Strong positive correlations were observed between plasma and serum concentrations for 17β‐estradiol (*r* = 0.72; *P *< 0.001) and progesterone (*r* = 0.89; *P *< 0.001). The mean bias and limits of agreement for plasma versus serum were 12.5 pg/ml (−20.6 to 45.5 pg/ml) for 17β‐estradiol and 1.01 ng/ml (−5.6 to 7.6 ng/ml) for progesterone. Ovarian hormone levels were consistently higher in EDTA plasma compared with serum, with these matrices not yielding statistically equivalent results. Despite these differences, the strong correlations and good agreement suggest that both matrices are suitable for biomarker analysis. Researchers using EDTA plasma should account for the higher hormone concentrations when applying inclusion or exclusion criteria, because adjustments might be necessary to ensure appropriate participant classification.

## Abstract (highlights)

- **What is the central question of this study?**
  The aim was to determine whether the concentrations of 17β‐estradiol and progesterone, as measured by immunoassay, differed between plasma and serum in young, physically active females.
- **What is the main finding and its importance?**
  EDTA‐treated plasma yields higher ovarian steroid hormone concentrations than serum. Researchers using plasma should account for the higher hormone concentrations to ensure appropriate inclusion/exclusion of participants and accurate classification of menstrual cycle status.

## 1 INTRODUCTION

The call to prioritize research on females in areas including medicine (O'Bryan et al., 2022), nutrition (Kuikman et al., 2023; Rogan & Black, 2023), psychology (Avila‐Varela et al., 2024), exercise physiology (Lew et al., 2022) and sport (Burden et al., 2024) continues to grow, with increasing recognition of the need to address the historical under‐representation of this population in studies. A common reason cited for exclusion of females from research is the complexity of accounting for the menstrual cycle or contraceptive use (Smith et al., 2022). Although studying females in natural, free‐living conditions might be more ecologically valid, accurately testing hypotheses related to sex hormones requires strict control of the menstrual cycle and contraceptive phases/use, which is costly, labour intensive and time consuming (Merrell et al., 2024). Currently, the consensus view is that the menstrual cycle phases and contraceptive use have minimal or no impact on various aspects of physiology in healthy, young and active females (D'Souza et al., 2023). However, these conclusions are confounded by studies adopting poor methodological approaches and lacking verification of hormonal status (D'Souza et al., 2023). Thus, there remains insufficient high‐quality data to draw definitive conclusions about the effects of cyclical fluctuations in endogenous estrogen and progesterone (McNulty et al., 2020) or the suppression of endogenous hormones through contraceptive use (Elliott‐Sale et al., 2020) on female exercise physiology and performance. As such, a personalized approach based on the response of an individual to exercise training across the menstrual cycle is currently recommended (McNulty et al., 2020).

Accurately confirming whether participants have a regular, ovulatory menstrual cycle, in addition to ensuring adequate levels and/or variations in hormone concentrations, is essential for drawing valid conclusions about potential variations in female health, physiological function and performance throughout the menstrual cycle (Elliott‐Sale et al., 2021). It also aids in differentiating between females with a healthy menstrual cycle and those experiencing menstrual dysfunction (Elliott‐Sale et al., 2021). This distinction is especially important when evaluating physically active women of reproductive age, because a significant number can face menstrual disturbances, such as luteal phase deficiency and anovulation (Manore, 2002), which can affect ovarian steroid profiles, despite women still presenting with a regular menstrual cycle. To this end, it is recommended to use a combination of calendar‐based tracking, urinary luteinizing hormone surge testing and serum measurements of 17β‐estradiol and progesterone to verify menstrual cycle status (Janse De Jonge et al., 2019). 17β‐Estradiol and progesterone are commonly assessed using commercially available immunoassay kits. To our knowledge, no studies have examined whether blood collection tube chemistries influence the measured concentrations of these sex hormones when using immunoassays, despite some available assays permitting the use of various biofluids (plasma and serum) with the same reference ranges. Serum‐based measurements are often preferred because the absence of clotting factors and other proteins provides a cleaner matrix for hormone analysis. However, if samples are not processed promptly, hormone degradation can occur in serum tubes. In such cases, EDTA plasma might be preferable, because it seems to tolerate short processing delays better than serum (Stevens et al., 2019). Thus, the aim of this study was to determine whether the concentrations of the ovarian hormones 17β‐estradiol and progesterone, as measured by immunoassay, differed between plasma and serum in young and physically active females.

## 2 MATERIALS AND METHODS

### 2.1 Participants

Recreationally active/trained females (*n *= 25) gave written informed consent to participate in this study, which was approved by Loughborough University Research Ethics Approvals Human Participants Sub Committee (ethics code: R19‐P137).

### 2.2 Study design

This investigation was a sub‐analysis of data collected as part of a larger, multipart study, and detailed study population characteristics are published elsewhere (Rowland et al., 2023). Briefly, we recruited 13 females with a regular, natural menstrual cycle (21–35 days in duration, with nine or more consecutive periods per year and evidence of a luteinizing hormone surge, without the use of any hormonal contraceptives within the last 6 months) (Elliott‐Sale et al., 2021). These participants attended the laboratory during two separate menstrual cycles and captured two distinct hormonal profiles: (1) the early follicular phase (days 1–4), i.e., low concentrations of 17β‐estradiol and progesterone; and (2) the mid‐luteal phase (the middle 4 days of the luteal phase, determined from the predicted cycle duration minus the day of ovulation, which was ∼7–9 days after a positive urinary luteinizing hormone test), i.e., high concentrations of 17β‐estradiol and progesterone. Females (*n *= 12) using 21‐day combined, monophasic oral contraceptive pills were also recruited. The pill brands included Rigevidon (*n* = 5), Lucette (*n* = 2), Levest (*n* = 2), Millinette (*n *= 1), Cilique (*n *= 1) and Lizinna (*n *= 1). Contraceptive users were tested twice during the off‐pill phase (days 1–4), i.e., when no exogenous synthetic hormones were ingested, and twice during the on‐pill phase (days 17–21).

### 2.3 Blood collection and analysis

After 30 min of supine rest, a tourniquet was applied to the upper arm, and venous blood was sampled from an antecubital vein via venepuncture into EDTA (K_2_) and gold serum separator tubes (SST) vacutainers (BD vacutainers, Medisave UK Ltd, UK). Plasma was centrifuged (3500*g* at 4°C for 10 min), extracted and stored at −80°C. The serum tube was left to clot for 15 min at room temperature before being centrifuged, aliquoted, then stored at −80**°**C. Plasma and serum 17β‐estradiol and progesterone concentrations were determined in duplicate using competitive immunoenzymatic assays in accordance with the manufacturer's instructions (Abcam, Cambridge, UK: ab108667 and ab108670, respectively). The intra‐assay coefficient of variation for 17β‐estradiol was 3.6% (serum) and 3.4% (plasma), with a detection limit of 8.68–2000 pg/ml, and for progesterone it was 2.4% (serum) and 3.0% (plasma), with a detection limit of 0.05–40 ng/ml.

### 2.4 Statistical analysis

Statistical analyses were conducted using GraphPad Prism v.10.1.2, with statistical significance accepted at *P *≤ 0.05. The Shapiro–Wilk test confirmed that the data were not normally distributed, and therefore correlations between plasma and serum concentrations were examined using Spearman's rank. Between methods (plasma vs. serum) differences were assessed using a Wilcoxon matched pairs signed rank test. Non‐parametric limits of agreement were examined using Bland–Altman plots to assess the closeness of agreement between measured concentrations in plasma and serum.

## 3 RESULTS

17β‐Estradiol concentrations were undetectable in 40 pairs of plasma and serum samples (early follicular phase, *n *= 6; mid‐luteal phase, *n *= 1; inactive pill phase, *n *= 15; and active pill phase, *n *= 18) and detectable but below the limit of quantification in 18 pairs (early follicular phase, *n *= 3; mid‐luteal phase, *n *= 1; inactive pill phase, *n *= 8; and active pill phase *n *= 6). Progesterone concentrations were above the upper limit of detection in six pairs (all mid‐luteal phase). Data analysis was conducted on the remaining 42 pairs for 17β‐estradiol and 94 pairs for progesterone (Table 1).

**TABLE 1:** 17β‐Estradiol and progesterone data included in method comparison analysis.

Parameter	Eumenorrhoeic females (*n *= 13)		Oral contraceptive users (*n *= 12)	
	Early follicular phase	Mid‐luteal phase	Pill withdrawal phase	Pill consumption phase
17β‐estradiol (pg/ml)				
Pairs included/ excluded	17/9	24/2	1/23	0/24
Serum, mean ± SD (range)	19.9 ± 5.8 (10.8–32.7)	49.7 ± 25.5 (12.5–127.7)	21.9	N/A
Plasma, mean ± SD (range)	29.4 ± 10.9 (12.5–50.1)	64.6 ± 28.4 (20.0–136.5)	25.6	N/A
Progesterone (ng/ml)				
Pairs included/ excluded	26/0	20/6	24/0	24/0
Serum, mean ± SD (range)	1.9 ± 2.2 (0.1–9.4)	21.0 ± 10.3 (5.8–38.8)	0.7 ± 0.4 (0.3–1.6)	0.7 ± 0.3 (0.2–1.5)
Plasma, mean ± SD (range)	1.8 ± 1.7 (0.2–9.1)	24.1 ± 10.5 (5.8–36.8)	1.4 ± 0.8 (0.3–3.9)	1.5 ± 0.9 (0.4–3.8)

*Note*: Each participant was tested twice during each respective phase, two pairs per person. Abbreviation: N/A, not available.

**FIGURE 1:**
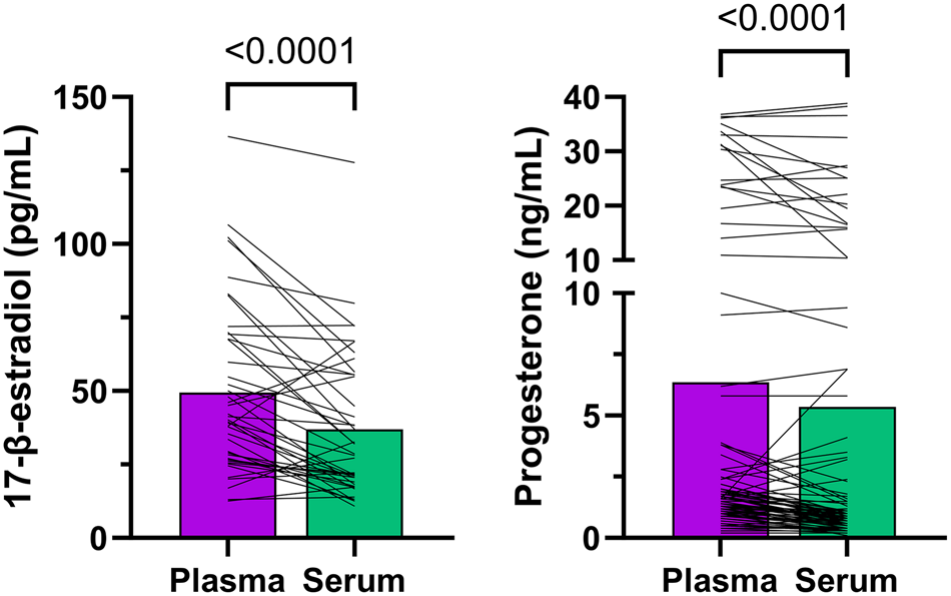
Comparison of plasma and serum 17β‐estradiol and progesterone concentrations. Bars represent the medians, and individual lines represent individual participants.

**FIGURE 2:**
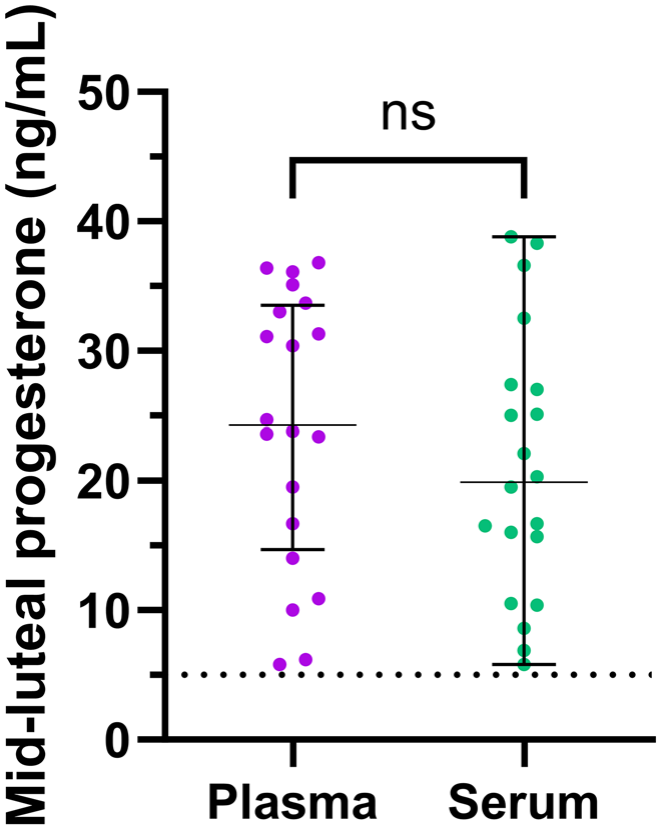
Mid‐luteal phase progesterone concentrations in plasma and serum. Data are presented as the median and 25th and 75th percentiles, with individual dots representing individual participants. The dashed line represents the serum luteal phase verification threshold. Abbreviation: ns, not significant.

**FIGURE 3:**
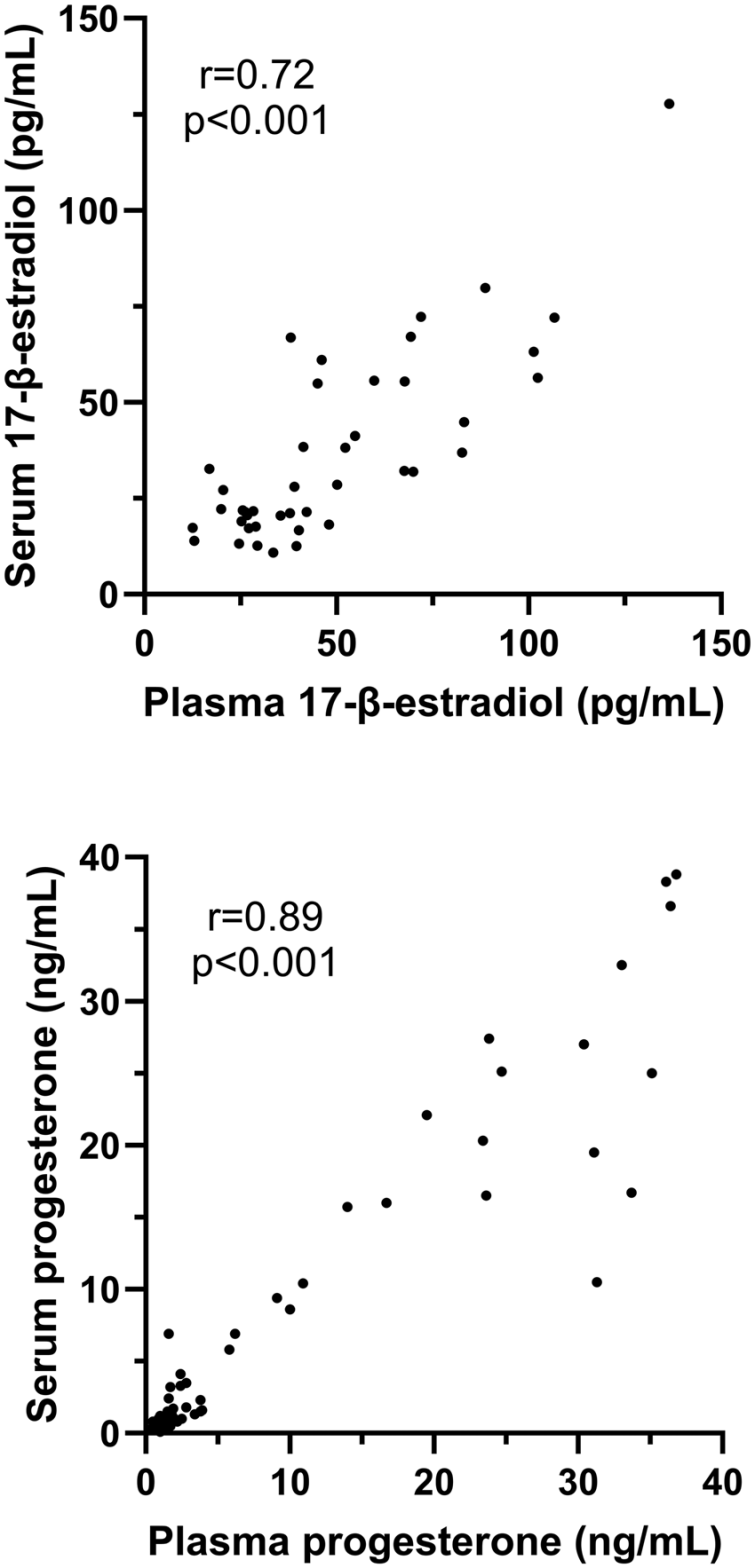
Scatter plots from Spearman's ρ analysis showing the correlation between plasma and serum concentrations for 17β‐estradiol (*r* = 0.72, *P *< 0.001) and progesterone (*r* = 0.89, *P *< 0.001).

**FIGURE 4:**
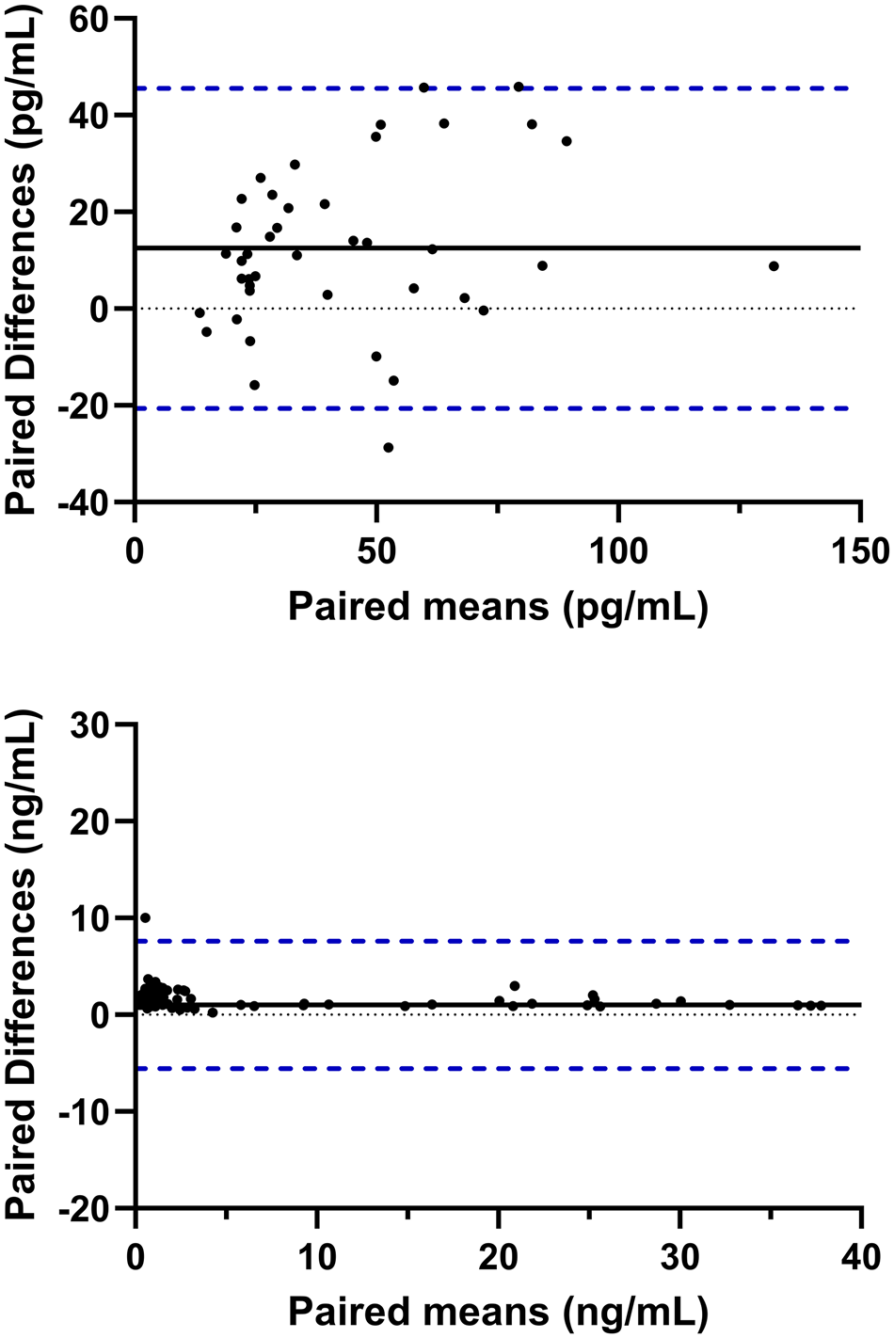
Bland–Altman plots showing the agreement between plasma and serum concentrations of 17β‐estradiol (paired means, in picograms per millilitre) and progesterone (paired means, in nanograms per millilitre). Mean paired difference (middle black line); estimated upper and lower limits of agreement (upper and lower blue lines) = 95th and 5th quantile, respectively. Grey dashed line visually represents 0 pg/ml (17β‐estradiol) and 0 ng/ml (progesterone).

Median plasma concentrations of 17β‐estradiol were 12.50 pg/ml higher than in serum [median (interquartile range) plasma 40.75 (27.00–68.10) pg/ml vs. serum 28.25 (18.80–55.45) pg/ml, *P *< 0.001] and 0.75 ng/ml higher than serum for progesterone [plasma 1.70 (1.00–3.80) ng/ml vs. serum 0.95 (0.50–3.65) ng/ml, *P *< 0.001; Figure 1]. In the menstrual cycle group, mid‐luteal progesterone levels in plasma [24.25 (14.68–33.53) ng/ml] were similar to those in serum [19.90 (11.80–27.30) ng/ml, *P* = 0.172], with all individuals exceeding the luteal phase verification threshold of >5 ng/ml regardless of collection tube chemistries (Figure 2). There was a strong positive correlation between plasma and serum concentrations for 17β‐estradiol (*r* = 0.72; *P *< 0.001) and progesterone (*r* = 0.89; *P *< 0.001; Figure 3). The mean bias and limits of agreement for plasma versus serum were 12.5 pg/ml (−20.6 to 45.5 pg/ml) for 17β‐estradiol and 1.01 ng/mL (−5.6 to 7.6 ng/ml) for progesterone (Figure 4).

## 4 DISCUSSION

These results show that ovarian hormone concentrations are generally higher in EDTA plasma than in serum, with 17β‐estradiol averaging 44.2% higher and progesterone 78.9% higher in EDTA plasma. Despite these differences, there were strong positive correlations and good agreement between the methods for both analytes. Although both serum and EDTA plasma are suitable for biomarker analysis, the higher concentrations in plasma should be considered when applying conservative inclusion or exclusion criteria. For instance, current guidelines recommend serum progesterone levels of >16 nmol/l (5 ng/ml) during the mid‐luteal phase to rule out luteal phase deficiency (Janse De Jonge et al., 2019). Notably, all menstruating women in this study were classified as eumenorrhoeic and did not exhibit mid‐luteal phase deficiency. Therefore, future research comparing progesterone concentrations across different blood collection methods in females with mid‐luteal phase deficiency would be a valuable addition in this field. Researchers using EDTA‐treated plasma might need to adjust this threshold accordingly to ensure appropriate inclusion/exclusion of participants and accurate classification of menstrual cycle status.

To advance research in the field of female physiology, further research is necessary to refine the methods for collecting and analysing commonly assessed sex hormones. First, most studies in sport and exercise science currently depend on immunoassays for measuring sex hormone concentrations in human biofluids. However, a key limitation of immunoassays is their potential cross‐reactivity with other similarly structured compounds (Ong et al., 2023). Therefore, it is crucial for users of these kits to review and assess the cross‐reactivity data before purchasing or using such assays. Mass spectrometry, recognized as the gold standard for steroid measurement owing to its superior specificity and accuracy in quantifying these metabolites, is recommended by clinical journals and could provide significant advantages in this field (Taylor et al., 2015), particularly for studies investigating sex hormone‐driven hypotheses. However, the need for specialist infrastructure and technical training and the difficulties in quantifying estradiol concentrations at low levels mean that this technique is not commonplace in laboratories at present (Ong et al., 2023). Second, venous blood sampling is the current gold standard for measuring ovarian hormones, but the invasive nature of this method presents a significant barrier to participation in research and data collection in field‐based settings. Therefore, an area worthy of investigation is the potential use of fingertip capillary blood sampling and/or saliva or urine collection for analysing 17β‐estradiol and progesterone levels. Third, there is considerable inter‐individual variability in sex hormone levels (Dam et al., 2022: see their supplementary material), resulting in broad and overlapping reference ranges across menstrual cycle phases (Elliott‐Sale et al., 2021). In addition, the extent of data variation within and between different immunoassay kit manufacturers is unknown. A limitation of the present study is evidence of proportional bias and heteroscedasticity in the progesterone data, potentially linked to the assay used. As a result, the 95% limits of agreement might not accurately reflect the true range of differences, particularly at extreme measurement values, and should, therefore, be interpreted with this limitation in mind. Further research is needed to evaluate data analysed using different methods and by various manufacturers to gain a better understanding of the potential for these forms of bias. Implementing a standardized analytical protocol would be valuable for assessing inter‐laboratory agreement and could enhance the comparability of studies examining homogeneous populations (e.g., athletes), a process known as laboratory harmonization (Plebani, 2018). One possible approach is for researchers to incorporate certified reference standards at relevant ranges for sex steroids during analysis. However, it is important to note that the cost of these reference standards might be prohibitive for some laboratories, potentially limiting widespread implementation.

## 5 CONCLUSION

In conclusion, our findings align with previous research showing strong agreement between 17β‐estradiol measurements in serum and plasma using liquid chromatography–tandem mass spectrometry (Coburn et al., 2019). Our study extends this work by using immunoassays, an alternative analytical technique commonly used in exercise science laboratories, and by examining an additional steroid hormone, progesterone. We found that hormone concentrations were higher in EDTA‐treated blood compared with serum, with these matrices not yielding statistically equivalent results. It remains unknown whether similar outcomes would be observed in heparinized or citrate‐treated plasma or across different commercially available immunoassay kits. Developing a broader understanding of how different blood collection tube chemistries affect hormone concentrations would be valuable for improving the accuracy and comparability of hormone measurements across studies. Finally, it remains to be determined whether hormone concentrations show strong agreement between venous blood and other biological samples, such as capillary blood, saliva and urine. Clarifying this would benefit both laboratory and field studies and help researchers to track acute changes throughout the menstrual cycle(s) and contraceptive ‘phases’, because these non‐invasive biofluids are easy to collect and do not require a trained phlebotomist.

## AUTHOR CONTRIBUTIONS

Samantha N. Rowland and Stephen J. Bailey conceived and designed the study. Samantha N. Rowland completed data collection. All authors were involved in the sample analysis and/or interpretation. Samantha N. Rowland wrote the first draft of the manuscript. All authors reviewed and/or edited the final version of the manuscript and agree to be accountable for all aspects of the work. All persons designated as authors qualify for authorship, and all those who qualify for authorship are listed.

## CONFLICT OF INTEREST

None declared.

## Data Availability

The raw data can be obtained on request.
